# A microcosting study of immunogenicity and tumour necrosis factor alpha inhibitor drug level tests for therapeutic drug monitoring in clinical practice

**DOI:** 10.1093/rheumatology/kew292

**Published:** 2016-08-29

**Authors:** Meghna Jani, Sean Gavan, Hector Chinoy, William G. Dixon, Beverley Harrison, Andrew Moran, Anne Barton, Katherine Payne

**Affiliations:** ^1^Centre for Musculoskeletal Research,; ^2^Arthritis Research UK Centre for Epidemiology, Institute of Inflammation and Repair,; ^3^Manchester Centre for Health Economics, Institute of Population Health, Manchester Academic Health Science Centre University of Manchester,; ^4^National Institute of Health Research Manchester Musculoskeletal Biomedical Research Unit, Central Manchester NHS Foundation Trust Manchester Academic Health Science Centre, Manchester; ^5^Department of Rheumatology, Salford Royal NHS Foundation Trust, Salford; ^6^Department of Immunology; ^7^Kellgren Centre for Rheumatology, Manchester Royal Infirmary, Central Manchester University Hospitals NHS Foundation Trust, Manchester, UK

**Keywords:** microcosting, immunogenicity, TNFi drug levels, opportunity costs, health economics

## Abstract

**Objectives.** To identify and quantify resource required and associated costs for implementing TNF-α inhibitor (TNFi) drug level and anti-drug antibody (ADAb) tests in UK rheumatology practice.

**Methods.** A microcosting study, assuming the UK National Health Service perspective, identified the direct medical costs associated with providing TNFi drug level and ADAb testing in clinical practice. Resource use and costs per patient were identified via four stages: identification of a patient pathway with resource implications; estimation of the resources required; identification of the cost per unit of resource (2015 prices); and calculation of the total costs per patient. Univariate and multiway sensitivity analyses were performed using the variation in resource use and unit costs.

**Results.** Total costs for TNFi drug level and concurrent ADAb testing, assessed using ELISAs on trough serum levels, were £152.52/patient (range: £147.68–159.24) if 40 patient samples were tested simultaneously. For the base–case analysis, the pre-testing phase incurred the highest costs, which included booking an additional appointment to acquire trough blood samples. The additional appointment was the key driver of costs per patient (67% of the total cost), and labour accounted for 10% and consumables 23% of the total costs. Performing ELISAs once per patient (rather than in duplicate) reduced the total costs to £133.78/patient.

**Conclusion.** This microcosting study is the first assessing the cost of TNFi drug level and ADAb testing. The results could be used in subsequent cost-effectiveness analyses of TNFi pharmacological tests to target treatments and inform future policy recommendations.

Rheumatology key messages

- Microcosting analysis enabled quantification of resource use and costs required to implement TNF inhibitor pharmacological monitoring in practice.
- The cost of £152.52/patient for TNF inhibitor pharmacological monitoring (base case analysis) was comparable to other novel diagnostics.
- The additional appointment for trough level TNF inhibitor pharmacological monitoring was the key driver of costs per patient.

## Introduction

TNF-α inhibitors (TNFi) have transformed the treatment of several chronic inflammatory diseases. Given their effectiveness in the most severely affected patients, the use of biologics in rheumatology continues to increase, but is associated with significant expenditure (£10 000/year/patient). TNFi agents such as adalimumab, etanercept and infliximab are currently represented within the top five highest medicinal expenditures in England [1], with an estimated cost to the National Health Service (NHS) of ∼£160 million annually for RA [2]. A targeted approach using robust predictive biomarkers of response in TNFi-treated patients may add value to the clinical decision-making process by potentially informing the selection of which TNFi drug to use first in specific patients, the appropriate biologic sequence and whether to continue the drug in patients established on therapy. However, there remain considerable gaps in the evidence base supporting the introduction of a targeted approach into clinics [3]. In the era of finite budgets, robust economic evidence is required in order to ensure that the alternative uses for funds are considered in any decision, and decision-making groups must be aware of other funding pressures and service developments that will otherwise be forgone (opportunity costs) [4].

An important mechanism for treatment failure of certain TNFi agents is immunogenicity involving the formation of anti-drug antibodies (ADAb) and low drug levels [5, 6]. While the presence of ADAbs and low TNFi drug levels, detected soon after treatment initiation, have been shown to predict subsequent treatment response [7], tests quantifying levels are not currently available in rheumatology clinical practice in the UK NHS. Such testing needs to be both effective in improving outcomes and a cost-effective use of the healthcare budget before it can be recommended for implementation into the clinic. To date, a description of the types and quantity of resources needed to provide the test is not available in the published literature. Identifying the resources required will facilitate the calculation of the costs of implementing these tests in a UK clinical setting if the introduction of such testing is shown to be clinically useful.

Microcosting is a method that allows robust assessment of the types and quantities of resources and associated costs of health interventions consumed [8]. It is particularly useful for estimating the costs of new interventions and for interventions with large variability across providers, thereby potentially providing a key input for undertaking subsequent economic evaluations. The aim of this study was to identify and quantify the resource use and associated costs required for introducing drug level and ADAb testing to assess response to TNFi drugs in routine practice in the UK setting.

## Methods

A microcosting study assumed the NHS (service provider) perspective for identifying the resource use and cost per patient of providing TNFi drug level and ADAb testing (the test). Costs of providing the test were determined from the point of a patient established on treatment (for ⩾3 months) presenting to clinic, to the results being fed back to the clinician to inform a treatment decision. Direct medical costs associated with providing the test were identified; indirect non-medical costs (such as absence from work) were not consistent with the study perspective and beyond the scope of the paper. Ethical approval was not required. This study was essentially an audit of practice in North West England. (Regional guidelines for biologics in RA [9] allow use of these tests in rheumatology practice if clinicians have access.) The four study stages are now described.

### Stage 1: identifying the testing pathway

**Fig. 1:**
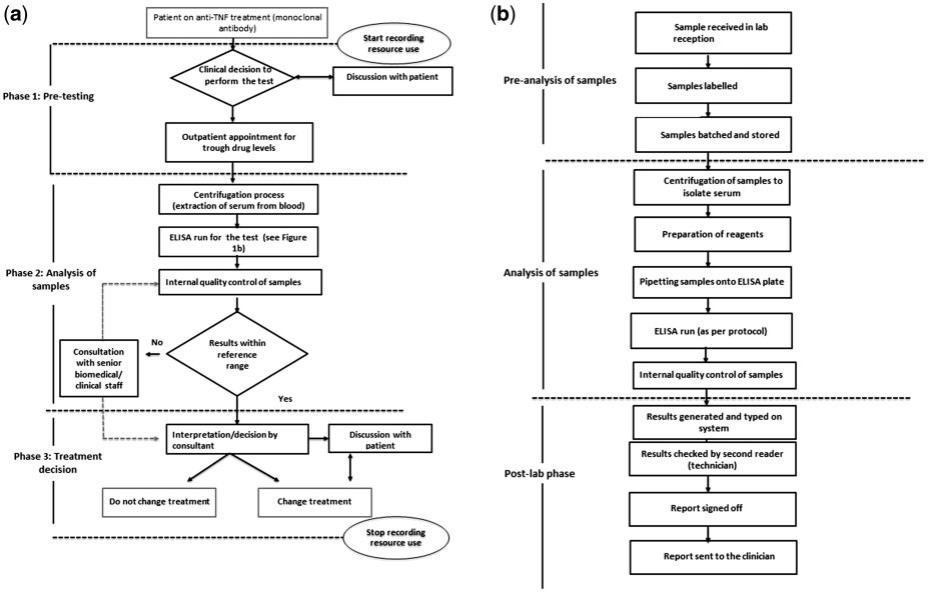
Pathway for immunogenicity and drug level testing (the test) (**A**) Overview of the pathway from the clinical decision to perform the test. (**B**) Summary of laboratory processes.

The test is not routinely available in UK rheumatology practice, and it was necessary to define an explicit pathway for a patient being offered testing with input from six experts from North West England (four rheumatology consultants and two clinical/laboratory staff) (Fig. 1A and B). The pathway eventually encompassed three phases: pre-testing, analysis of samples, and treatment decision (Fig. 1A). This study assumed that the test is reliant on identifying a pre-defined drug trough level, requiring an additional outpatient appointment, rather than using random sampling, which mirrors the current availability of the technology available in the UK (bridging ELISAs) to measure ADAbs.

### Stage 2: use of resources

The use of direct medical resources for the pathway was estimated using structured face-to-face interviews and elicitation with six experts. The expert elicitation process is described in more detail in supplementary Table S1, available at *Rheumatology* Online. Direct non-participant observation of staff was undertaken in a hospital setting to generate an estimate of the time taken for selected procedures. The Central Manchester Foundation NHS Trust Immunology Department was asked to name resources required relating to laboratory staff time. The level of each resource use was estimated per patient for each phase and per batch of 40 samples for the laboratory processes (Fig. 1A, phase 2).

### Stage 3: identifying unit costs

It was assumed that most hospital laboratories would have the necessary room requirements and stock standard equipment required to perform ELISAs, and the following items of resource use were therefore excluded: equipment costs of centrifuge systems; ELISA readers; pipettes; personal protective equipment; phlebotomy equipment costs; overhead; and capital costs.

A variety of approaches were taken to identify unit costs (price year 2015) for each type of resource use (Table 1). The unit cost of a rheumatology blood-monitoring appointment was obtained from operational managers for rheumatology directorates of two hospitals (Central Manchester University Hospitals and Salford Royal NHS Foundation Trust). Published estimates of unit costs for labour time were not available for all types of staff by the Personal Social Services Research Unit [10]. Therefore hospital-based health care staff time was valued using relevant labour unit costs from the national pay system for the NHS (Agenda for change—pay rates 2015–16) [11] and the British Medical Association pay scale for medical staff in England (2015–16) [12]. Salary scales per annum were converted to a per-minute rate by dividing the number of workable minutes per year, as described previously [13] (see supplementary Table S2, available at *Rheumatology* Online).

**Table 1:** Resource use and costs of implementing drug level and immunogenicity testing per patient in a hospital setting

Type of resource use	Staff member	Mean volume of resource use, minutes	Range of resource use, minutes	Source to obtain resource use	Unit costs for base-case analysis 2015, £^a^	Range of unit costs 2015, £	Source to obtain unit costs [reference]	Total costs (£) (minimum to maximum range)
**Phase 1: pre-testing**								
Outpatient appointment for discussion about need for test	Consultant rheumatologist	3.6 min	2–5 min	Expert estimation	£39.24/h	£33.42–45.06/h	BMA pay scales [12]	£2.35 (£1.11–3.76)
Clerical staff (to book the appointment and send out a letter to patient)	Clerical assistant	8.2 min	6–11 min	Direct observation	£8.43/h	£7.72–9.12/h	NHS pay scale, band 5 [11]	£1.15 (£0.77–1.67)
Appointment for trough blood levels	Phlebotomist/ clinical support worker	Resource use incorporated in unit cost	NA	NA	£102 per appointment	NA	DOH national tariff [14]	£102 (NA)
**Phase 2: analysis of samples**								
Receipt and labelling of samples—central specimen reception^b^	Medical lab assistant	15 min	NA	Expert estimation	£8.86/h	£7.74–9.98/h	NHS pay scale band 5 [11]	£2.22 (£1.94–2.50)
Data entry of patient information to lab system^b^	Medical lab assistant	15 min	NA					£2.22 (£1.94–2.50)
Sample preparation—extraction of serum from blood^b^	Medical lab assistant	15 min	NA					£2.22 (£1.94–2.50)
Transport, receipt and storage of sample—immunology lab^b^	Medical lab assistant	15 min	NA					£2.22 (£1.94– 2.50)
Preparation of reagents (wash solution, setting up assay, conjugate)^b^	Biomedical scientist	15 min	NA	Expert estimation	£12.79/h	£11.12–14.45/h	NHS pay scale band 5 [11]	£3.20 (£2.78–3.61)
ELISA kit for ADAbs or drug levels^b^	NA	NA	NA	NA	£700.00 per ELISA	NA	UK commercial price from Grifols^c^	£700.00
Pipette tips for ELISAs^b^	NA	NA	NA	NA	£6.00 per ELISA	NA	University of Manchester Centre for Musculoskeletal Research laboratory	£6.00
Semi-deep well plates for ELISAs^b^	NA	NA	NA	NA	£2.20 per ELISA	NA	Laboratory costs	£2.20
Troughs for ELISAs^b^	NA	NA	NA	NA	£1 per ELISA	NA	Laboratory costs	£1.00
Retrieval of patient/IQC samples from storage^b^	Biomedical scientist	10 min	NA	Expert estimation	£12.79/h	£11.12–14.45/h	NHS pay scale- band 5 [11]	£2.13 (£1.85 –2.40)
Checking and sorting samples to match worklist^b^	Biomedical scientist	10 min	NA					£2.13 (£1.85–2.40)
Pipetting samples onto ELISA plate^b^	Biomedical scientist	20 min	NA					£4.26 (£3.71– 4.81)
Pipetting calibrators, IQC samples and incubation of samples^b^	Biomedical scientist	10 min	NA					£2.13 (£1.85 –2.40)
Washing ELISA plate and addition of conjugate^b^	Biomedical scientist	10 min	NA					£2.13 (£1.85 –2.40)
Washing ELISA plate and addition of substrate^b^	Biomedical scientist	10 min	NA					£2.13 (£1.85–2.40)
Addition of stop solution^b^	Biomedical scientist	5 min	NA					£1.06 (£0.93 –1.20)
ELISA plate reading and printing of results^b^	Biomedical scientist	10 min	NA					£2.13 (£1.85–2.40)
Technical validation involving review of Internal quality control^b^	Biomedical scientist	5 min	NA					£1.06 (£0.93–1.20)
Results transcribed to worksheet^b^	Biomedical scientist	5 min	NA					£1.06 (£0.93–1.20)
Data entry of results to patient record in lab system^b^	Biomedical scientist	10 min	NA					£2.13 (£1.85 – 2.40)
Transcribed results/data entry reviewed by a second independent biomedical scientist^b^	Biomedical scientist	5 min	NA					£1.06 (£0.93 – 1.20)
Clinical authorisation using reference range/delta check failure results^b^	Senior Biomedical scientist or Consultant immunologist	5 min	NA	Expert estimation	£30.48/h	£15.9–45.06/h	NHS pay scale band 7 assumed [11]; BMA pay scale [12]	£2.54 (£1.33 –3.76)
Hardcopy report sent to clinician^b^	Clerical assistant	15 min	NA	Expert estimation	£8.43/h	£7.74 –9.12/h	NHS pay scale [11]	£2.11 (£1.93 –2.28)
**Phase 3: Treatment decision**								
Interpretation of results by rheumatologist	Consultant Rheumatologist	6 min	4–10	Expert estimation	£39.24/h	£33.42–45.06/h	NHS pay scale [11]	£3.92 (£2.23 –7.51)
Discussion with patient (phone call)		5.3 min	5–6					£3.47 (£2.79–3.92)
Letter with results and decision		3.3 min	3–4					£2.16 (£1.61–2.62)
**Total costs (best case to worst case scenario)^d^**								£152.52 (£147.68–159.24)

a Mid-point of salary grade used to calculate base–case sample.

b Resource use estimated per batch (40 samples).

c Previously known as Progenika Biopharma.

d Base–case (multiway sensitivity analyses were conducted by varying the following parameters using pre-defined lower and upper ranges of estimated resource use: lowest time taken to perform tasks using the lowest pay grade (best case scenario) and highest amount of time taken to perform procedures using the highest pay grade (worst case scenario). BMA: British Medical Association;

DOH: department of health; ELISA: enzyme-linked immunosorbent assay; IQC: internal quality control; NA: not applicable; NHS: National Health Service.

### Stage 4: data analysis

The base–case analysis calculated the total cost of the test by multiplying unit costs with the identified items and quantities of resource for each phase of the pathway (see Fig. 1A). Multiway sensitivity analyses were conducted by varying the following parameters using lower and upper ranges of estimated resource use: lowest time taken to perform tasks using the lowest pay grade (best case scenario) and highest amount of time taken to perform procedures using the highest pay grade (worst case scenario). Three one-way sensitivity analyses and one two-way sensitivity analysis were used to understand the impact of varying pre-defined assumptions made when calculating the cost of the test.

## Results

Table 1 summarizes the items and quantity of resource use and unit costs for each of the three phases of the pathway (Fig. 1A).

### Base–case analysis

The total cost for performing the test was £152.52/patient for the base–case analysis. The most expensive element of the pathway was the cost of the additional appointment to conduct blood sampling for drug trough levels. Therefore the pre-testing phase incurred the highest costs due to the additional appointment to perform trough blood sampling (total costs: £105.50/patient). The total cost for processing 40 samples during laboratory phase (phase 2, analysis of samples) was £749.34 [£18.73 (cost in phase 2 divided by 40) × 2 (for both tests) = £37.47/patient to simultaneously perform the test]. The final treatment decision cost was £9.55/patient. The additional trough level appointment accounted for 67% of the total cost, and labour and consumables accounted for 10% and 23% of the total costs, respectively.

### Sensitivity analyses

The multiway sensitivity analysis varied the estimated and directly observed time and pay grade for each phase (see Table 1). Using the lowest values, the estimated best-case scenario was £147.68/patient/test. Using the highest values, the worst-case scenario estimated a cost of £159.24.

Three one-way sensitivity analyses were performed (see Sensitivity analysis in the supplementary data, available at *Rheumatology* Online). Performing the tests singly and not in duplicate may reduce test accuracy, but lowered the total cost to £133.78/patient. If the patient was due to take their TNFi on the day following their rheumatology appointment, an additional trough level appointment was not required, lowering the test cost to £50.52. If there were 50 samples to be processed by the laboratory, a new batch would need to be started, increasing the resource use in phase 2 and the total cost to £173.79/patient.

One two-way sensitivity analysis examined the impact of using various pay grades. For costs attributed to consultant time (base–case), varying the pay scale to the lower grade using the mean volume of resource use (Table 1) changed the total costs to £145.26/patient. The variation in grade included a specialty trainee in rheumatology at £38 588.50/annum (mid-point of paygrade, supplementary Table S2, available at *Rheumatology* Online), a consultant rheumatologist (Table 1, phases 1 and 3) and a senior clinical biochemist (mid-point of paygrade £35 891/annum, supplementary Table S2, available at *Rheumatology* Online) instead of a consultant immunologist (Table 1, phase 2).

## Discussion

This microcosting study has identified the potential direct medical costs associated with TNFi pharmacological testing from a service provider’s perspective in the UK. Since these tests for TNFi-treated patients are not routinely performed in UK clinical practice, a testing pathway was developed to allow a detailed estimation of the quantities of resources required in order to calculate a total cost. The developed pathway provides a framework for reporting resource use, presenting unit costs and allowing decision-makers from various jurisdictions to use their country-specific data if required.

There is accumulating evidence that monoclonal TNFi drug levels and ADAb levels correlate with future response to the drugs [7, 15]. If the testing strategy is to translate to clinical practice, a number of points will need to be addressed. First, the test must be shown to be useful in changing clinical decision-making; second, robust evidence must confirm that the change in practice will result in better outcomes for patients; finally the test intervention should be a cost-effective use of health care budgets. The current work is the first step in informing the last requirement. To date, the costs associated with providing TNFi drug level/ADAb testing are not known because no national tariff exists for diagnostic tests. Emerging numbers of microcosting studies in other areas have enabled rigorous comparison of health interventions in order to inform efficient resource allocation [16]. A recent NICE diagnostic assessment committee evaluating test performance of ELISA kits for ADAbs and TNFi levels in Crohn’s disease was not able to draw definitive conclusions about the relative cost-effectiveness of the test compared with current practice because of insufficient evidence to inform the analysis. Early analysis suggested that the test may save the NHS money, but would also result in some loss of health in the population tested. The high degree of uncertainty in the economic analysis, particularly around the impact of the test on quality-adjusted life-years meant that the committee concluded that further research was required before the test could be recommended for use in clinical practice [17].

Our microcosting analysis identified a unit cost of £152.52/patient, making this biomarker test for guiding decisions regarding future treatment with TNFi comparable with that of other novel diagnostics and theranostics [18]. A robust economic evaluation that identifies the incremental costs and health benefits (quality-adjusted life-years) of using the test for targeting TNFi treatments compared with current prescribing practice in RA is required to determine whether this targeted approach is a cost-effective use of health care budgets.

The overall cost of testing per patient in the UK was influenced most by the cost of an additional appointment for obtaining trough levels. When the cost of trough levels was excluded, the cost per patient reduced to £50.52. To deal with batching and capacity, the base–case analysis assumed batching of samples from 40 patients/ELISA. However, uneven sample numbers would require a new batch with changes in marginal costs (cost of doing one more test) impacting on consumables, staff resources and time. If results are to be fed back in sufficient time for referring clinicians to make treatment decisions, it is unlikely that samples from 40 patients would be available for testing unless test sites were restricted to regional or national laboratories. When processing 50 rather than 40 samples, the cost per sample rose to £173.79 because each ELISA kit only allows for 40 samples to be analysed at a time.

This analysis made several assumptions in order to estimate the total cost. The base–case assumed a tertiary level setting in the north-west of England; however, it is acknowledged that the cost of a trough level appointment may vary elsewhere in the UK, thus influencing the total costs/sample. We assumed a concurrent testing strategy for all samples, in which tests for TNFi drug levels and ADAbs were performed at the same time, rather than reflex testing, which may be an alternative to reduce costs. Reflex testing would involve testing the TNFi drug levels first and only testing for ADAbs if the drug was undetectable. Direct non-medical costs such as patient out-of pocket expenses for trough level testing were not included, which may have wider societal implications [19]. Capital/overhead costs were not included in the analysis. While some hospital laboratories have automated ELISA systems or multiplex platforms, this was not assumed and was deemed unlikely to significantly lower resource estimates, following consultation with the hospital laboratory team. Furthermore, while numerous ELISA kits are commercially available for ADAb and TNFi drug level testing, unit costs were based on those ELISA kits frequently used in the literature [20].

In conclusion, using a microcosting approach, we have explicitly identified and quantified the types and quantities of resources required in order to provide TNFi drug and ADAbs level testing in an NHS clinical setting and found that the costs were comparable with those of other tests already available. The identified cost of the test will be of use for future cost-effectiveness analysis of TNFi pharmacological testing. The results of the study will also help inform potential resource implications per patient for hospital trusts considering incorporating pharmacological monitoring into clinical practice.

## Supplementary Material

Supplementary Data
