# Supplementary material for: A microcosting study of immunogenicity and tumour necrosis factor alpha inhibitor drug level tests for therapeutic drug monitoring in clinical practice
Source: Rheumatology (Oxford). 2016 Aug 29;55(12):2131–7. doi: 10.1093/rheumatology/kew292 (PMC5144665; doi:10.1093/rheumatology/kew292)
Supplement: Supplementary Data [file supp_55_12_2131__index.html]

A microcosting study of immunogenicity and tumour necrosis factor alpha inhibitor drug level tests for therapeutic drug monitoring in clinical practice — A microcosting study of immunogenicity and tumour necrosis factor alpha inhibitor drug level tests for therapeutic drug monitoring in clinical practice — Supplementary Data 

# A microcosting study of immunogenicity and tumour necrosis factor alpha inhibitor drug level tests for therapeutic drug monitoring in clinical practice

## Supplementary Data

files

- Supplementary Data - docx file
