# Supplementary material for: A microcosting study of immunogenicity and tumour necrosis factor alpha inhibitor drug level tests for therapeutic drug monitoring in clinical practice
Source: Rheumatology (Oxford). 2016 Aug 29;55(12):2131–7. doi: 10.1093/rheumatology/kew292 (PMC5144665; doi:10.1093/rheumatology/kew292)
Supplement: Supplementary Data [file supp_kew292_rhe-15-1860-File004.docx]

**Supplementary Data**

**Sensitivity analyses**

The following sensitivity analyses were performed assessing: the lowest paygrade; performing the tests singly rather than in duplicate; not requiring a trough level sample; and if 50 samples were tested instead of 40. For the first sensitivity analysis assuming a rate of £15.46/hour for specialty trainee in rheumatology and £18.40/hour for senior biomedical scientist, overall cost was £145.26 per patient (Phase 1= £104.08; Phase 2= 18.71*2 = £37.42; Phase 3= £3.76). For the second analysis performing the tests singly rather than in duplicate, resulted in an overall cost of £145.26 per patient (Phase 1= £104.08, Phase 2= 18.71*2 = £37.42, Phase 3= £3.76). For the third sensitivity analysis, not requiring a trough level reduced the overall cost to £50.52 per patient [Phase 1= £3.50 (105.50-102 {cost of blood monitoring appointment}), Phase 2= 37.47, Phase 3= £9.55)]. If 50 samples were tested instead of 40, the overall cost would increase to £173.79 per patient (Phase 1 = £105.50, Phase 2 = £58.74, Phase 3 = £9.55). Supplementary Table S3 outlines phase 2 costs for the fourth sensitivity analysis.

**Supplementary Table S1: Expert elicitation study design**

|  | Description |
| --- | --- |
| 1. Rationale for expert elicitation | Since pharmacological monitoring of TNFi agents is not used currently in routine UK clinical practice expert elicitation was used to generate estimates which were used in the microcosting analysis |
| 1. Research problem | The uncertain quantities estimated by expert elicitation were as follows:  Time during an outpatient appointment for discussion about need for the tests  Analysis of samples (Phase 2, Table 1)  Treatment decision (Phase 3, Table 1) |
| 1. Measure type of uncertain quantities | Time for each activity |
| 4. The expert panel (and numbers of experts) | -The expert panel comprised of four consultant rheumatologists with an interest in inflammatory arthritis and some knowledge of the potential utility of the test in clinical practice  - Two clinical and laboratory staff provided estimated for the steps in phase 2 (one who had laboratory experience of performing the outlined tests using the Grifols kits referenced in this analysis and the other with extensive experience performing ELISAs in a hospital laboratory setting) |
| 5. Preparation | The protocol used for expert elicitation became the template used for Table 1 and figure 1a and 1b |
| 6. Piloting | The first person interviewed during the expert elicitation provided the pilot for subsequent interviews |
| 7. Data collection and exercise | Data collection was obtained from semi-structured face-to face interviews, which were guided by a set of open and closed questions (n=7) to determine time estimates outlined |
| 8. Administration | Mode of administering the expert elicitation was using a structured data collection form |
| 9. Training | No training materials were used as experts selected were highly familiar with topic |
| 10. Data recording | Initially transcribed using pen and paper, followed by an excel spread sheet |
| 11. Data aggregation | Individual responses were aggregated by calculating the mean estimated value |
| 12. Ethical issues | No ethical approval was necessary for this study as it was deemed to be an audit of practice in the North West of England (Greater Manchester Medicines Management Group Guidelines for biologics in RA [1] allow use of testing in rheumatology practice if clinicians have access to immunogenicity and drug level testing) |
| 13. Presentation of results | The point estimate and distribution for each of the uncertain quantities are presented in Table 1. |

**Supplementary Table S2: Staff pay-scales 2015-16**

| **Title** | **Pay scale (£)** | **Working time assumed (hours per week)** |
| --- | --- | --- |
| **Consultant^a^** | 75,249 - 101,451 | 43.3 |
| **Specialist registrar** | 30,002 - 47,175 | 48 |
| **Clerical assistant** | 15,100 - 17,800 | 37.5 |
| **Medical lab assistant** | 15,100 - 19,461 | 37.5 |
| **Clinical support worker (band 2-3)** | 15,000 - 19,461 | 37.5 |
| **Biomedical scientist (band 5)** | 21,692 - 28,180 | 37.5 |
| **Senior Biomedical scientist (band 7)** | 31,072 - 40,964 | 37.5 |

^a^Based on 2003 contract in England (excluding potential clinical excellence awards).

**Supplementary Table S3: Sensitivity analysis if 50 samples tested (phase 2 costs)**

| **Process** | **Resource required** | **Cost (£)** | **Total cost of process (£)** |
| --- | --- | --- | --- |
| Receipt and labelling of samples – central specimen reception | 18.75 | 8.86 | 2.77 |
| Data entry of patient information to lab system | 18.75 | 8.86 | 2.77 |
| Sample preparation – extraction of serum from blood | 18.75 | 8.86 | 2.77 |
| Transport, receipt and storage of sample – immunology lab | 18.75 | 8.86 | 2.77 |
| Preparation of reagents (wash solution, setting up assay, conjugate) | 18.75 | 12.79 | 4.00 |
| ELISA kit for ADAbs or drug levels | NA | 700 | 1400 |
| Pipette tips for ELISAs | NA | 6 | 12 |
| Semi-deep well plates for ELISAs | NA | 2.2 | 4.4 |
| Troughs for ELISAs | NA | 1 | 2 |
| Retrieval of patient/IQC samples from storage | 12.5 | 12.79 | 2.66 |
| Checking and sorting samples to match worklist | 12.5 | 12.79 | 2.66 |
| Pipetting samples onto ELISA plate | 25 | 12.79 | 5.33 |
| Pipetting calibrators, IQC samples & incubation of samples | 12.5 | 12.79 | 2.66 |
| Washing ELISA plate and addition of conjugate | 12.5 | 12.79 | 2.66 |
| Washing ELISA plate and addition of substrate | 12.5 | 12.79 | 2.66 |
| Addition of stop solution | 6.25 | 12.79 | 1.33 |
| ELISA plate reading and printing of results | 12.5 | 12.79 | 2.66 |
| Technical validation involving review of Internal quality control | 6.25 | 12.79 | 1.33 |
| Results transcribed to worksheet | 6.25 | 12.79 | 1.33 |
| Data entry of results to patient record in lab system | 12.5 | 12.79 | 2.66 |
| Transcribed results/data entry reviewed by a second independent biomedical scientist | 6.25 | 12.79 | 1.33 |
| Clinical authorisation using reference range/delta check failure results | 6.25 | 30.48 | 3.18 |
| Hardcopy report sent to clinician | 18.75 | 8.43 | 2.63 |
|  | **Total Cost of Phase 2** | £1468.59 | |
|  | **Total cost per sample** | £29.37 | |
|  | **Total cost for both tests** | £58.74 | |

NB: For 50 samples - assumption (a) Per ELISA Costs are doubled, (b) Time resource input increased by 25%

**References**

[1] Jani M, Reid V, Parker B, et al. Harmonised Biologics Pathway for Rheumatoid Arthritis. Greater Manchester Medicines Management/ MAHSC Guidelines. http://gmmmg.nhs.uk/docs/guidance/GMMMG RA Pathway 22 april_2015.pdf
